# How you get it matters: Moderating role of transfer method in guiding ownership assignments for modified objects

**DOI:** 10.3389/fpsyg.2022.957079

**Published:** 2023-01-10

**Authors:** Zhanxing Li, Dong Dong

**Affiliations:** ^1^School of Humanities and Social Sciences, Institute of Social Psychology, Xi’an Jiaotong University, Xi’an, China; ^2^Department of Mechanical Science and Bioengineering, Graduate School of Engineering Science, Osaka University, Toyonaka, Japan

**Keywords:** ownership, labor, transfer, property, creation

## Abstract

**Introduction:**

Previous research has found that value change and creation drive people to support the laborer more than the original owner in ownership reasoning for modified objects; however, the transfer methods used to resolve conflicts have largely been ignored. In this work, two studies were designed to investigate the role of value change and creation in adults’ labor-based ownership judgments in four transfer conditions (i.e., *take/steal/borrow/find*).

**Methods:**

Scenarios involving different extent of value change and creation in different transfer ways were presented to Chinese adult subjects after which they were asked to judge who is the owner.

**Results:**

People were more likely to assign ownership to the original owner in the *take*, *steal* and *borrow* conditions but not in the *find* condition, and this reasoning held regardless of whether the original materials showed high or low value appreciation or successful creation, and it was applicable to raw materials with low (Study 1) and high values (Study 2). In addition, the effect of value change and creation on ownership reasoning varied according to different transfer methods.

**Conclusion:**

The results suggest the moderating role of transfer method in people’s ownership assignments, which will provide insights for real-life mediation of ownership conflicts.

## 1. Introduction

We are living in a world that involves property and ownership. Ownership is an important social institution that maintains human relationships (Epstein, 1978; Kanngiesser et al., 2016), and it is a concept used to protect individual property rights and respect the property rights of others (Kanngiesser et al., 2019, 2020). As the idiom goes, *possession is nine-tenths of the law*. Thus, many interpersonal conflicts are incurred by ownership problems. Studies have empirically explored the preferential resolution strategies of laymen when confronted with these problems (Friedman, 2008; Friedman and Neary, 2008, 2009; DeScioli and Karpoff, 2015; Fast et al., 2016, 2017; DeScioli et al., 2017) and found that their resolutions were not always consistent with the legal provisions (Fast et al., 2016, 2017) or final verdict in court (DeScioli and Karpoff, 2015; DeScioli et al., 2017), which suggests that legal judgments regarding certain property issues may not correspond to the psychological expectations of laypeople. Knowing the psychological mechanisms that people use to resolve ownership disputes may shed light on real-life ownership trials and help reduce the gap between legal decisions and laymen’s judgments.

When we intervene in property issues, labor is an important cue for resolving conflicts. According to Locke’s property theory, the work of a man’s body and his hands should be seen as rightfully owned by him (Locke, 1690/1978). Psychological research supports that both young children and adults tend to use labor rules to attribute ownership (Beggan and Brown, 1994; Kanngiesser and Hood, 2014a; Levene et al., 2015; Hartley et al., 2021). For example, 2–3 year-olds would protest when someone threatened to take away their homemade clay animals but would not protest when the original materials were taken away (Kanngiesser and Hood, 2014a; Hartley et al., 2021). In a study conducted by Beggan and Brown (1994), adults were more inclined to assign ownership to an agent when the agent modified a discovered branch into a plane than when he just played with the branch. Palamar et al. (2012) also revealed that people were more likely to assign ownership to the man who intentionally knocks a pineapple from a tree rather than the man who picks it up on the ground afterward. Occasionally, ownership disputes arise between the original owner of a material and the modifier of the material. Imagine a piece of wood originally owned by someone that is then modified by another person into a set of furniture. Should we assign ownership of the furniture to the modifier or the original owner of the wood? Some scholars addressed this question with British 4-year-old children and adults (Kanngiesser et al., 2010). First, they gave subjects a piece of clay as their own and asked the subjects to make animal models out of the clay provided to the other subjects. The results showed that most preschoolers acknowledged that ownership transferred to the model maker, but many adults did not make the such assumption. Cross-cultural research showed that Japanese adults were more likely than British counterparts to transfer ownership to the laborers when they saw the third-party conflicts between the initial owners and the modifiers (Kanngiesser et al., 2014). However, another study revealed that Chinese adults supported the original possessor rather than the modifier as owner (Li et al., 2019). It can be seen that there is cultural difference in ascribing ownership based on the labor rule. Some studies also showed that adults give priority to the laborers over the original creators when it comes to the intellectual property (Fast et al., 2016, 2017; Burgmer et al., 2019). For example, they tend to ascribe ownership of an artwork to the person who actually made it than to the person who came up with the idea (Burgmer et al., 2019) and hold that the alteration should be permissible if the laborer has acknowledged the original creator of the artwork (Fast et al., 2016, 2017).

Scholars have proposed that value change might be an underlying mechanism for people to reason about ownership with the labor rule as ownership and value often go together (Pesowski et al., 2022). Previous research showed that there is a labor-valuation effect in people’s preferential conflict resolution (Burgmer et al., 2019). For example, when a laborer put another person’s idea into practice which makes the final artwork high-valued, people often allocate more money to the laborer than to the idea giver (Burgmer et al., 2019). Kanngiesser and Hood (2014b) presented adult subjects with scenarios in which an artist takes some materials from another man and modifies them into artwork, and they found that people were more likely to transfer ownership when the artist’s labor greatly increased the value of the original materials but less likely to do so when the value changed little. In addition, the subjects were more inclined to transfer ownership for low-value materials (i.e., plastic) than for the high-value materials (i.e., gold).

Creation is another important element that people will refer to when making ownership judgments on the grounds of labor cue (Kanngiesser et al., 2010, 2014; Levene et al., 2015; Faigenbaum et al., 2018; Davoodi et al., 2020). Children and adults often regard others as having stronger claims over things they created than things they found (Casiraghi et al., 2018; Faigenbaum et al., 2018; Davoodi et al., 2020). Kanngiesser et al. (2010) showed that children would transfer ownership to the laborers when the modifier creatively made an animal model out of the original owners’ clay, but they would not when the laborers just cut off a small piece of the clay using a knife. The same result was found in a cross-cultural study (Kanngiesser et al., 2014). Levene et al. (2015) established a scenario in which a man either crushed a can into an ashtray with a rock (creation condition) or just dented it without changing its function (no creation condition), and the ashtray or dented can was finally picked up by another man. The authors found that adult subjects were more likely to attribute ownership to the laborer when creation was involved, thus suggesting that creation will affect people’s support for the laborer.

Most of the aforementioned studies did not explicitly discriminate among the transfer methods when the laborer obtained the original materials from the original owners. People may obtain others’ objects through illegal (e.g., steal) or legal (e.g., borrow) ways and process them, which result in a great increase in value and creative change, thereby cause complex property conflicts. In Kanngiesser and Hood’s (2014b) study, they depicted a scenario in which the artist *take*s the materials from the original owner. While *take* is often used as a legitimacy-neutral verb, we did not know whether the artist’s taking behavior was permitted by the original owners. People’s ownership assignments may change if the modifying behavior is not approved by the original owner or the raw materials are obtained in some illegal way (such as *steal*). Previous studies have shown that people would not transfer ownership for the stolen objects and the lost objects, but they would permit ownership transferred or in the gift-giving context (Blake and Harris, 2009; Li et al., 2018; McDermott and Noles, 2018). Hook (1993) explored people’s ownership assignments when someone *borrowed* another person’s wood and modified it into a falcon without permission. They found that children above 10 years old and adults would not support the modifier as the owner of the falcon, even if the value of the wood had appreciated greatly. The same result was also found in Chinese adult samples (Li et al., 2019).

Recently, Li et al. (2020) examined Chinese adults’ ownership assignments when faced with disputes between the original owner and the modifier in three transfer contexts (i.e., *keep*, *borrow* and *find*). In the *keep* context, the original owner keeps his wood in the modifier’s house and the modifier modifies them into a set of furniture without permission. In the *borrow* context, the wood was borrowed from the original owner by the modifier. In the *find* context, the wood was lost by the original owner and found by the modifier. These authors found that subjects tended to support the original owner as owner of the furniture in the *keep* context but tended to support the modifier in the *find* context; however, the subjects did not support either the original owner or the modifier in the *borrow* context. In the study, the authors did not discriminate the degree of value change (e.g., large value change, small value change) in the scenarios. While the modification from woods to furniture can be regarded as a large increase in value, we do not know how people will attribute ownership when the value change is small. In addition, the study selected only one kind of raw material (woods) in the scenarios; however, the initial value of the raw material may affect the subjects’ ownership assignments, as Kanngiesser and Hood (2014b) discovered.

### 1.1. Current study

In summary, previous studies have acknowledged the role of value change and creation in people’s ownership resolution between the original owner and the modifier, but most of the studies examined the role of the two independently. Value change and creation may affect people’s ownership judgments in interactive ways. For example, one person may transform another person’s cup into a sound box such as to make a creative change, but the price of the cup and the sound box may be in similar level. Alternatively, people may simply deal with an item without creatively changing it, but will increase its value. As a case in point, people often give higher value to items (e.g., shirts) worn or used by celebrities, even if the shirts have not been changed during the historical course (Huang et al., 2017). It is worth testing how will people solve ownership problems when pitting value change against creation for us to understand the relative importance of different cues in ownership representation.

Moreover, fewer studies have explored how the context in which a modifier obtains raw materials affects the subjects’ ownership judgments. Due to the different legitimacy of the transfer methods, people’s ownership judgments may be swayed in different extent when the modified objects have different value added or creative changes. People are unlikely to transfer ownership for stolen objects even if the processing has made them appreciate greatly because it is commonly seen as a serious illegal behavior to steal others’ items. But people may permit ownership transferred for the found objects due to relatively neutral description and that people have a tendency to assign responsibility to the original owners for losing objects (Li et al., 2020). They are also likely to permit ownership transferred for the borrowed objects by attributing that the lenders did not declare beforehand that any processes are not allowed for the original objects. The permissions are more likely to occur when the laborers make the original objects gain great value compared to less value.

To address these questions, we comprehensively investigated the role of value change, creation and transfer method in people’s labor-based ownership judgments. The results will help us uncover the relative importance of ownership cues in resolving property conflicts and provide insights into the methods of settlement in reality. We performed two studies to investigate Chinese adults’ preferential resolutions for modified objects with low initial value (Study 1) and high initial value (Study 2) with a scenario-based method, as many previous studies have used (e.g., Kanngiesser and Hood, 2014b; Li et al., 2020). The value change (high, low) and creation (with/no creation) of an object and transfer methods were set in the scenarios. With regard to value change, a laborer processes an original owner’s object which causes a large or small change in its value. With regard to creation, a laborer transforms an original owner’s object into a new one thus changing its function (creation condition), or just adds some traces on it without changing its original function (no creation). This refers to previous studies (e.g., Levene et al., 2015) since people are ready to link creation with functional changes (Judge et al., 2020). We selected four transfer methods in the study, i.e., *steal, borrow, find,* and *take*. The *steal* condition was selected because stealing is an obviously illegal behavior and the taking behavior is not permitted according to common sense. Even three-year-old children would deny that ownership was transferred in such a context (Blake and Harris, 2009; Li et al., 2019). The *borrow* condition was selected because the taking behavior is often approved in this context although the modifying behavior may not allowed by the lender. The *find* condition was selected because in this context, the finder may not know that the discovered objects were originally owned and the objects were inadvertently modified. Finally, we set a *take* condition because it has no clear license meaning or legitimacy meaning and this would offer us a baseline for comparisons with the results from other three conditions and that of a previous study (i.e., Kanngiesser and Hood, 2014b).

As with previous studies, we expected that there would be significant effect of value change in subjects’ ownership assignments such that they would be more likely to transfer ownership in the large value change condition than in the small value change condition, and there would be significant effect of creation in subjects’ ownership assignments such that they would be more likely to transfer ownership in the creation condition than in the no creation condition. In addition, we expected that there would be significant effect of transfer method in subjects’ ownership assignments such that they would be more likely to transfer ownership in the *find* condition than in other three conditions as previous research showed (Li et al., 2020). Especially, we expected that interactive effects might be found between the transfer method and value change and between the transfer method and creation. Due to the obvious illegality of theft, people are unlikely to transfer ownership even if the processing has made the stolen goods gain greatly added value or creative change. But for the conditions of *borrow* and *find*, people’s ownership assignments may sway with the value change and creation.

## 2. Study 1

### 2.1. Subjects

We used G*Power 3.1 statistical software to estimate the sample size. *A priori* power analysis indicated that to reach a medium effect *f*  = 0.25, alpha = 0.05, power = 0.80, the study needed to include 120 participants. Finally, we recruited 148 college students (age range: 17 ~ 26 years old, *M*_age_ = 20.07, *SD* = 1.05, 61 males) from a university in Northwest China as subjects, with the aim to include a sufficient sample size given the possibility of invalid responses. Subjects learned about the study through recruitment posters and contacted us voluntarily by cell phone or other network communication tools. We excluded students majoring in law to avoid the possible effect of professional knowledge background in ownership judgments. The study was conducted with the approval of the Scientific Research Ethics Committee of our unit. All subjects provided signed informed consent before participation in the experiment.

### 2.2. Materials and procedure

Subjects were invited to the laboratory and asked to complete the study online. They were asked to read some scenarios and answer the questions after each scenario. The scenarios included materials that were originally owned by an agent but transferred to another agent in different ways, i.e., *take/steal/borrow/find*. Then, the new possessor of the material modified it into a new object, thereby greatly increasing the value (large value change condition) or only increasing the value to a small degree (small value change condition). Previous scholars have associated functional change with creation (e.g., Levene et al., 2015). Therefore, in this study, the modified object was depicted to have a new function in the creation condition while retaining the original function in the no creation condition. We selected three objects (i.e., a plastic cup, wood, and clay) that have relatively low values as the raw materials and implemented 48 scenarios that included the transfer mode (4), value change (2), creation (2) and object (3) as within-subjects factors. The following are the sample scenarios of the plastic cup.

#### 2.2.1. Take—large value change—creation condition

Li Ming has a plastic cup. Zhao Lei takes this cup and modifies it into a sound box. The initial price of the cup is ¥10,^1^ while the price is estimated to be ¥10,000^2^ after Zhao Lei modifies it into a sound box. Li Ming learns that the cup has been modified and claims the sound box. Finally, Li Ming and Zhao Lei quarrel about who owns the sound box.

#### 2.2.2. Steal—small value change—creation condition

Li Ming has a plastic cup. Zhao Lei steals this cup and modifies it into a sound box. The initial price of the cup is ¥10, while the price is estimated to be ¥11 after Zhao Lei modifies it into a sound box. Li Ming learns that the cup has been modified and claims the sound box. Finally, Li Ming and Zhao Lei quarrel about who owns the sound box.

#### 2.2.3. Borrow—large value change—no creation condition

Li Ming has a plastic cup. Zhao Lei borrows this cup and draws a picture on it. The initial price of the cup is ¥10, while the price is estimated to be ¥10,000 after Zhao Lei draws a picture on it. Li Ming learns that the cup has been modified and claims the cup. Finally, Li Ming and Zhao Lei quarrel about who owns the cup.

#### 2.2.4. Lose—small value change—no creation condition

Li Ming loses a plastic cup. Zhao Lei finds this cup and draws a picture on it. The initial price of the cup is ¥10. It’s estimated to be ¥11 after Zhao Lei draws a picture on it. Li Ming learns that the cup has been modified and claims the cup. Finally, Li Ming and Zhao Lei quarrel about who owns the cup.

For the other two raw materials, the wood was depicted as modified into a falcon model and the clay was depicted as molded into an animal figure (e.g., a Mickey Mouse figurine) in the creation condition. In the no-creation condition, Chinese characters are written on the wood^3^ while the clay was just kneaded into an oval shape by the laborer. Across all three raw materials, the value was set to change from ¥10 to ¥10,000 in the large value change condition and from ¥10 to ¥11 in the small value change condition. The names of the protagonists of different scenarios were varied to ensure that the subjects believed that they were reading different stories.

After each scenario, the subjects were first asked to answer how the laborer got the object, the extent of the value change and whether the object changed to another item to ensure that they understood and did not mix the content of the stories. Finally, the subjects were asked to provide their own opinions on who should own the modified objects by choosing from two options, i.e., the original owner of the raw materials and the modifier. They were instructed: “Please make decisions on ground of your own opinion and do not consider outside sources such as legal, economic, or political knowledge”. To control for possible spillover effects, scenarios corresponding to the *take* condition were always presented first because subjects’ responses in the other three conditions might prime the answer in this condition. For scenarios with the other three transfer conditions, the presenting order was counterbalanced. The presenting order of value change and creation were also counterbalanced. The total duration of the experiment is about 15 min.

### 2.3. Results

All subjects correctly answered the content understanding questions. Subjects’ choices were assigned a value of 1 if they selected the original owner as the owner. In contrast, they were assigned a value of 0 if they selected the modifier as the owner. A preliminary analysis showed that there were no significant differences across the three kinds of raw materials in ownership scores. Therefore, the ownership scores of three raw materials were summed up to form a composite score. The total score would be 3 if they selected the original owners in all three kinds of materials, and would be 0 if they selected the modifiers in all three kinds of materials. The midpoint of the ownership scores would be 1.5 [(0 + 3)/2] which represents the chance level of subjects’ ownership assignments.

A three-factor repeated measures analysis of variance (ANOVA) was conducted, with transfer type (4), value change (2) and creation (2) as the within-subjects independent variables. The results showed a significant main effect of transfer type (Greenhouse–Geisser test) [*F*(3, 441) = 80.02, MSE = 1.32, *p* < 0.001, η*_p_*^2^ = 0.35], with ownership scores ordered from the *steal* condition > *borrow* condition > *take* condition > *find* condition, and significant differences between each type of transfer (*p*s < 0.001) with the exception of that between the *steal* condition and the *borrow* condition (*p* = 0.796). The effect of value change was significant [*F*(1, 147) = 24.89, MSE = 0.98, *p* < 0.001, η*_p_*^2^ = 0.15]. Ownership scores were significantly higher in the small value change condition (*M* = 2.43, *SE* = 0.05) than in the high value change condition (*M* = 2.23, *SE* = 0.06). The effect of creation was significant [*F*(1, 147) = 26.54, MSE = 0.62, *p* < 0.001, η*_p_*^2^ = 0.15]. Ownership scores in the no creation condition (*M* = 2.41, *SE* = 0.05) were significantly higher than in the creation condition (*M* = 2.25, *SE* = 0.06).

**Figure 1:**
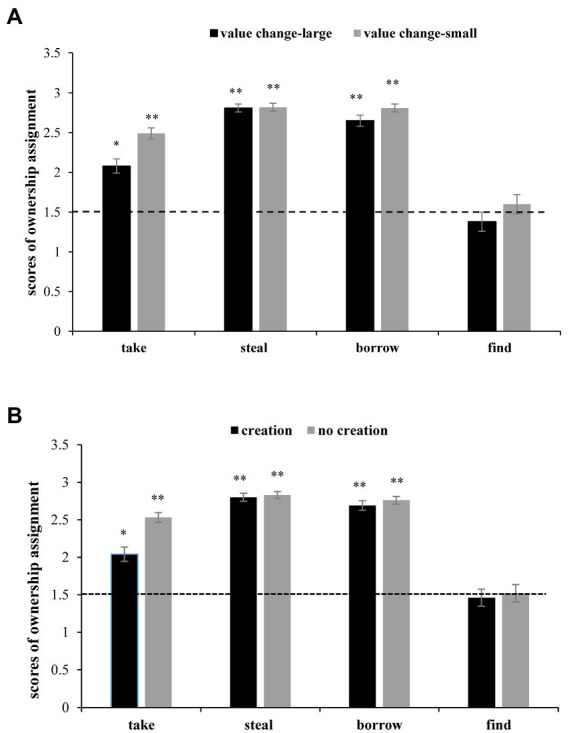
Ownership scores in different transfer conditions in Study 1. **(A)** Transfer type × value change interaction; and **(B)** transfer type × creation interaction. Note: *p < 0.05, **p < 0.01.

The interaction between transfer type and value change was significant [*F*(3, 441) = 9.31, MSE = 0.16, *p* < 0.001, η*_p_*^2^ = 0.06]. A simple effect analysis showed that the effect of transfer type revealed above held true for both the large and small value change conditions [*F*(3, 441) = 80.02, *p* < 0.001; *F*(3, 441) = 4.84, *p* = 0.003]. The effect of value change was significant in the *take* condition [*F*(1, 147) = 28.46, *p* < 0.001], *borrow* condition [*F*(1, 147) = 7.32, *p* = 0.008], and *find* condition [*F*(1, 147) = 10.19, *p* = 0.002] but not in the *steal* condition [*F*(1, 147) = 0.19, *p* = 0.660; see Figure 1A].

The interaction between transfer type and creation was also significant [*F*(3, 441) = 23.16, MSE = 0.14, *p* < 0.001, η*_p_*^2^ = 0.14]. A simple effect analysis revealed that under the creation condition and no creation condition, the transfer type effect was significant [*F*(3, 441) = 80.02, *p* < 0.001; *F*(3, 441) = 22.04, *p* < 0.001]. The effect of creation was significant only in the *take* condition [*F*(1, 147) = 40.72, *p* < 0.001] but not in the other three conditions [*steal*: *F*(1, 147) = 1.42, *p* = 0.236; *borrow*: *F*(1, 147) = 3.62, *p* = 0.059; *find*: *F*(1, 147) = 2.18, *p* = 0.142]. The interaction between value change and creation and the interaction between the three factors are not significant [*F*(1, 147) = 2.55, MSE = 0.08, *p* = 0.113, η*_p_*^2^ = 0.02; *F*(3, 441) = 0.11, MSE = 0.02, *p* = 0.957, η*_p_*^2^ = 0.00] (see Figure 1B).

We probed subjects’ favor to the original owners or the modifiers with one-sample *t-test*s by comparing their ownership scores to the midpoint in each condition. The results showed that in the *find* condition, the ownership scores corresponded to the chance level (*p*s > 0.05) regardless of whether the value change was large or small and whether creation occurred. In contrast, in the other three conditions, the ownership scores were significantly above the chance level (*p*s ≤ 0.002).

## 3. Study 2

Study 1 investigated how people would resolve ownership disputes when the original materials had low initial values. In Study 2, we investigated this issue using original materials with high initial values.

### 3.1. Subjects

One-hundred thirty-eight Chinese undergraduates (age range: 17.70 ~ 33.01 years old, *M*_age_ = 20.71, *SD* = 1.76, with 46 males) from our university participated in this study. All subjects were asked to provide signed informed consent before they participated in the experiment. Students who majored in law were excluded from this study.

### 3.2. Materials and procedure

The original materials in Study 1 were replaced with a gold bullion, rosewood and a diamond. Before the study, 60 subjects were asked to evaluate the initial value of these materials and the materials of Study 1 to ensure that the initial values were very different. The subjects were asked to rate the value of each material on a five-point scale from *extremely invaluable* (1) to *extremely valuable* (5). The results showed that the materials in Study 2 (gold bullion, *mean* = 4.08, *SD* = 0.11; rosewood, *mean* = 4.20, *SD* = 0.13; and diamond, *mean* = 4.00, *SD* = 0.13) were rated as more valuable than the materials in Study 1 (plastic cup, *mean* = 3.15, *SD* = 0.12; wood, *mean* = 3.12, *SD* = 0.13; clay, *mean* = 2.50, *SD* = 0.12, *p*s < 0.001), and rated as having a significantly higher value than the level of chance (3) (*p*s < 0.001). Due to the obviously high initial value, the price of the original materials was set to ¥10 thousand in this study, and the added value reached ¥10 million after modification in the large value change condition but ¥1.1 thousand in the small value change condition. The value changes were designed to correspond with those in Study 1. In the creation condition, the gold bullion was modified into a boat model, the rosewood was modified into a set of furniture, and the diamond was modified into a ring. In the no-creation condition, a picture was painted on the gold bullion; Chinese characters were written on the rosewood; and an oval shape was chiseled into the diamond. Thus, 48 scenarios were generated, which was consistent with Study 1, and the transfer mode (4), value change (2), creation (2) and object (3) were used as within-subjects factors. The following scenarios were presented.

#### 3.2.1. Take—large value change—creation condition

Li Ming has a gold bullion. Zhao Lei takes this gold bullion and modifies it into a boat model. The initial price of the gold bullion is ¥10 thousand, but it is estimated to be ¥10 million after Zhao Lei modifies it into a boat model. Li Ming learns that the bullion has been modified and claims the boat model. Finally, Li Ming and Zhao Lei quarrel about who owns the boat model.

#### 3.2.2. Steal—small value change—creation condition

Li Ming has a gold bullion. Zhao Lei steals this gold bullion and modifies it into a boat model. The initial price of the gold bullion is ¥10 thousand, but it is estimated to be ¥1.1 thousand after Zhao Lei modifies it into the boat model. Li Ming learns that the bullion has been modified and claims the boat model. Finally, Li Ming and Zhao Lei quarrel about who owns the boat model.

#### 3.2.3. Borrow—large value change—no creation condition

Li Ming has a gold bullion. Zhao Lei borrows this gold bullion and paints some pictures on it. The initial price of the gold bullion is ¥10 thousand, but it is estimated to be ¥10 million after Zhao Lei paints some pictures on it. Li Ming learns that the bullion has been modified and claims the gold bullion. Finally, Li Ming and Zhao Lei quarrel about who owns the gold bullion.

#### 3.2.4. Lose—small value change—no creation condition

Li Ming loses a gold bullion. Zhao Lei finds this gold bullion and paints some pictures on it. The initial price of the gold bullion is ¥10 thousand, but it is estimated to be ¥1.1 thousand after Zhao Lei paints some pictures on it. Li Ming learns that the bullion has been modified and claims gold bullion. Finally, Li Ming and Zhao Lei quarrel about who owns the gold bullion.

As with Study 1, scenarios in the *take* condition were always presented first. The presenting order of the other three transfer types, value change type and creation type were counterbalanced. The subjects were first asked to answer how the laborer got the object, the extent of the value change and whether the object changed to another item. Then they were asked to provide their own opinions according to their intuition on who should own the modified objects and choose from the original owners and the laborers. The total duration of the experiment is about 15 min. The participants were assigned a score of 1 if they selected the original possessors as the owner and a score of 0 if they selected the laborer as the owner.

### 3.3. Results

All subjects correctly answered the content understanding questions. Preliminary analysis indicates that there were no significant differences across the three kinds of raw materials in ownership scores. Composite scores were generated for further analysis. A repeated-measures ANOVA (4 transfer types × 2 value changes × 2 creation modes) was conducted with transfer type, value change and creation mode as the within-subject variables. The results revealed that the main effect of transfer type was significant [*F*(3, 411) = 52.06, MSE = 1.56, *p* < 0.001, η*_p_*^2^ = 0.28], with ownership scores ordered as steal condition > borrow condition > take condition > find condition, and significant differences were observed between each condition (*p*s ≤ 0.011). The effect of value change was significant [*F*(1, 137) = 37.73, MSE = 0.92, *p* < 0.001, η*_p_*^2^ = 0.22]. Ownership scores in the small value change condition (*M* = 2.32, *SE* = 0.05) were significantly higher than those in the large value change condition (*M* = 2.07, *SE* = 0.06). The effect of creation was significant [*F*(1, 137) = 26.14, MSE = 0.36, *p* < 0.001, η*_p_*^2^ = 0.16]. Ownership scores in the no creation condition (*M* = 2.26, *SE* = 0.05) were significantly higher than those in the creation condition (*M* = 2.13, *SE* = 0.06).

**Figure 2:**
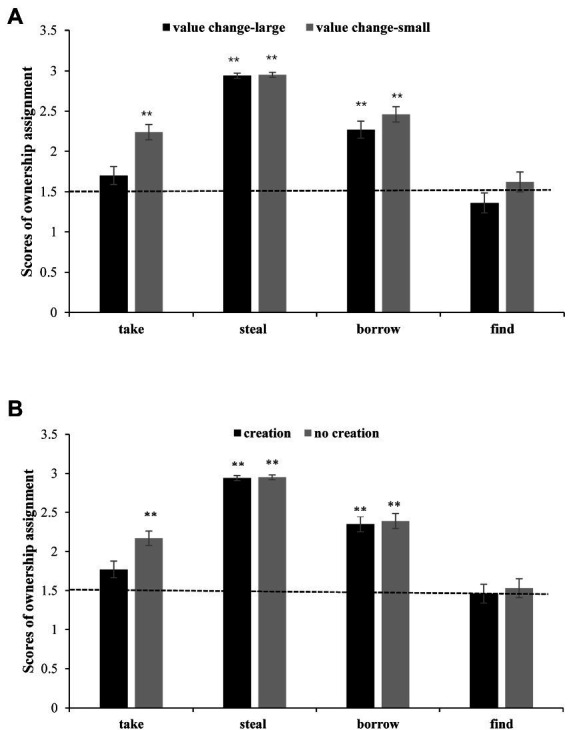
Ownership scores in different transfer conditions in Study 2. **(A)** Transfer type × value change interaction; and **(B)** transfer type × creation interaction. Note: *p < 0.05, **p < 0.01.

The interaction between transfer type and value change was significant [*F*(3, 411) = 14.36, MSE = 0.20, *p* < 0.001, η*_p_*^2^ = 0.16]. A simple effect analysis revealed that significant transfer type effects (*p*s < 0.001) occurred in the large and small value change conditions. The value change effect was significant in the *take*, *borrow* and *find* conditions (*p*s ≤ 0.002) but was not significant in the *steal* condition (see Figure 2A).

The interaction between transfer type and creation was also significant [*F*(3, 411) = 21.64, MSE = 0.14, *p* < 0.001, η*_p_*^2^ = 0.14]. A simple effect analysis revealed that the transfer type effect was significant (*p*s < 0.001) in the creation condition and the no creation condition. The creation effect was significant in the *take* condition (*p* < 0.001) and *find* condition (*p* = 0.022) but not significant in the *steal* condition and in the *borrow* condition. No other significant main effects or interactions were found. The interaction between value change and creation and the interaction between the three factors are not significant [*F*(1, 137) = 0.23, MSE = 0.02, *p* = 0.879, η*_p_*^2^ = 0.00; *F*(3, 411) = 1.20, MSE = 0.01, *p* = 0.308, η*_p_*^2^ = 0.01] (see Figure 2B).

One-sample *t tests* revealed that ownership scores were at the level of chance in the *find* condition, regardless of whether the value change was large or small or successful creation occurred. Ownership scores were also at the level of chance in the take*—*large value change*—*creation condition. Ownership scores were above the level of chance in other conditions (*p*s < 0.001).

Finally, we integrated the data from the two studies and tested the effect of the materials’ initial value through a mixed-design analysis of variance, with the initial value (high or low) as the between-subject variable and transfer type, value change, and creation as the within-subject variables. Similar to the results for Study 1 and in Study 2, the mixed-design results yielded a significant main effect of transfer type [*F*(3, 852) = 122.68, MSE = 1.37, *p* < 0.001, η*_p_*^2^ = 0.30], value change [*F*(1, 284) = 61.86, MSE = 0.95, *p* < 0.001, η*_p_*^2^ = 0.18], and creation [*F*(1, 284) = 51.07, MSE = 0.50, *p* < 0.001, η*_p_*^2^ = 0.15]. In addition, a marginally significant main effect of initial value was found [*F*(1, 284) = 3.64, MSE = 5.80, *p* = 0.057, η*_p_*^2^ = 0.01]. Ownership scores were higher for the low initial value materials (*M* = 2.33, *SE* = 0.05) than for the high initial value materials (*M* = 2.19, *SE* = 0.05). Transfer type was found to significantly interact with the value change [*F*(3, 852) = 23.65, MSE = 0.18, *p <* 0.001, η*_p_*^2^ = 0.08], creation [*F*(3, 852) = 44.05, MSE = 0.14, *p < 0*.001, η*_p_*^2^ = 0.13], and initial value of materials [*F*(3, 852) = 4.84, MSE = 1.37, *p* = 0.002, η*_p_*^2^ = 0.02]. A simple effect analysis showed that the transfer type effect was significant for materials with a low and high initial values (*p*s < 0.001). Ownership scores were significantly higher for materials with low initial values than high initial values in the *take* (*p* = 0.007) and *borrow* conditions (*p* = 0.001) but were significantly higher for materials with high initial values than low initial values in the *steal* condition (*p* = 0.029). No significant difference was found in the find condition (*p* = 0.978).

## 4. Study 3

In the above two studies, we found that subjects did not consider ownership to be transferred in the *take* condition. This finding seems to be inconsistent with Kanngiesser and Hood’s (2014b) study, which found that an adult would transfer ownership to the modifier as laborer when the original materials’ value appreciated greatly with modification. One possibility is that subjects’ ownership judgments would still be affected by the other three conditions because subjects were allowed to revise their answers, although the initial presented scenarios were in the *take* condition. To address this issue, we conducted a third study in which we extracted from the above two studies the scenarios with the *take* condition and presented them solely to subjects. Such a design would make it impossible for subjects’ ownership judgments in the take condition to be affected by other conditions.

### 4.1. Subjects

Ninety-six additional Chinese undergraduates (age range: 18.98 ~ 25.35 years old, *M*_age_ = 22.14, *SD* = 0.98, 8 males) were recruited as subjects in this study. They provided signed informed consent before participation.

### 4.2. Materials and procedure

Twenty-four scenarios corresponding to the *take* condition in Study 1 and Study 2 were extracted and integrated to form the materials in this study. The presentation order of these materials was counterbalanced according to the value change and creation conditions. The subjects were assigned a score of 1 if they selected the original possessor as the owner and a score of 0 if they selected the laborer as the owner.

### 4.3. Results

**Figure 3:**
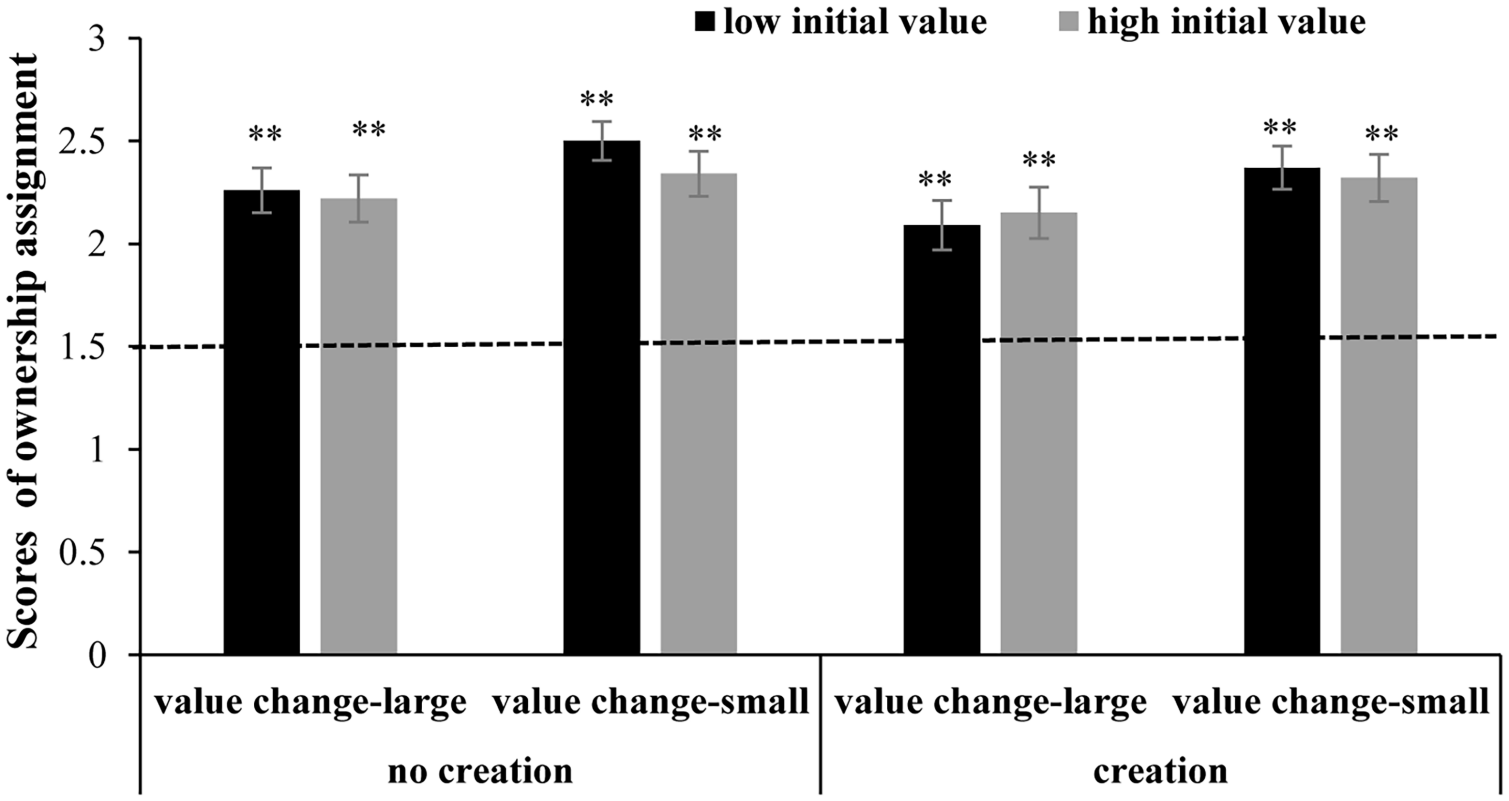
Subjects’ ownership scores in Study 3. Note: **p < 0.01.

Significant differences in ownership scores were not observed across the three kinds of raw materials in the preliminary analysis. A repeated-measures ANOVA (2 initial values × 2 value changes × 2 creation modes) was conducted with the transfer type, value change and creation as the within-subjects variables. The results showed that the effect of the initial value was not significant [*F*(1, 95) = 0.75, MSE = 0.63, *p* = 0.389, η*_p_*^2^ = 0.008]; the effect of creation was not significant [*F*(1, 95) = 1.79, MSE = 1.00, *p* = 0.185, η*_p_*^2^ = 0.02]; and the effect of value change was significant [*F*(1, 95) = 9.30, MSE = 0.87, *p* = 0.003, η*_p_*^2^ = 0.09]. Ownership scores in the small value change condition were significantly higher than those in the large value change condition. None of the interactions were significant (*p*s > 0.05). A one-sample *t test* showed that the ownership scores were significantly higher than chance (1.5) in all eight conditions (*p*s < 0.001) (see Figure 3).

## 5. Discussion

This study examined how people would resolve ownership disputes between the original owner and a laborer when the original materials were transferred in different ways and modified. By pitting value change and creation against transfer type, the results showed that the transfer method could affect ownership assignments. Subjects were more likely to support the original possessor as owner in the *take*, *steal* and *borrow* conditions, whereas they did not support the original owner in the *find* condition. The effect was significant whether the original materials showed high or low appreciation in value and whether successful creation was observed. This outcome is applicable to raw materials with low (Study 1) and high values (Study 2). Especially, we found transfer method interacts significantly with value change and creation in people’s ownership judgments. Subjects’ support for the original owners is significantly reduced in the *take*, *borrow* and *find* conditions when the modified objects appreciated greatly, but it remains unchanged in the *steal* condition. Subjects’ support for the original owners is significantly reduced in the *take* condition (and in the *find* condition, in Study 2) when there is successful creation, but it remains unchanged in the *steal* condition and in the *borrow* condition in two studies.

The study replicated results of previous research that value change would influence people’s ownership reasoning based on labor rule (Hook, 1993; Kanngiesser and Hood, 2014b; Burgmer et al., 2019), but there is also difference because previous research (e. g., Kanngiesser and Hood, 2014b) showed that people would transfer ownership to the laborers in the take condition but we did not found this in current study. This result held regardless of whether the value of the raw materials presented high or low changes and whether successful creation was observed. This finding was not due to the influence of the other three conditions because it held even when the scenarios in the *take* condition were presented independently (in Study 3). One possible reason for this finding is that Asian people are more conservative in judging ownership with regard to labor cues. For example, Kanngiesser et al. (2015) found that when asking British, Japanese and Chinese 4-year-olds to resolve ownership questions between an agent who obtained a piece of paper first and another agent who took it and painted a picture on it, British children were more likely to assign the picture to the painter while Japanese and Chinese children did not show such a tendency. In addition, they found that British adults favored the laborer more than the initial possessor while Japanese adults showed the inverse pattern (Chinese adults were not selected as subjects in this study). Future research should investigate the cultural factors that lead Asian adults to judge ownership more cautiously when labor cues are involved.

Previous research has revealed that creation will affect adults’ ownership resolution between the laborer and the subsequent possessor (Levene et al., 2015). This study demonstrates that creation will also play a role in solving conflicts between the laborer and the original owner. In addition, we found that both value change and creation interacted with transfer type in the study, and the interactive effects were similar between the two since whether a great value addition or the successful creation is not enough for subjects to change their ownership judgements for objects acquired in illegal way (i.e., *steal*), but will shake the extent of support for the original owners or the laborers when the acquisition method is relatively neutral (i.e., *take*, *find*). Although value increase does not necessarily lead to creation and creation may not lead to value appreciation, it may be easy for subjects to associate the two to make judgments in the same way because they are often concomitantly changed in most cases in life. Such explanation is worth considering because this study did not find the interaction between value change and creation. Alternatively, value change and creation may have a common foundation in guiding human’s ownership decisions. For example, people will think that both value appreciation and creation will make objects very different from their original state. Future research should examine these possible explanations further.

This study revealed that people consistently supported the original owners as owners of the newly made objects in the steal and borrow contexts, and the ownership scores in the *steal* condition and in the *borrow* condition were significantly higher than those in the *take* condition and in the *find* condition. The result suggests that people may not automatically represent taking behavior as stealing or borrowing because ownership scores in the *take* condition were significantly lower than those in these two conditions. This finding warns that previous studies only investigating people’s ownership opinions in the *take* context is not sufficient to reach a conclusion and we should distinguish among different transfer modes when exploring adults’ support for the original owner and the modifier. Interestingly, we found subjects’ ownership scores in the *find* condition were at the level of chance in both studies, which contrasts with Li et al. (2020)’s study that showed people would support the modifiers in this condition. It should be noted that there are three differences between this study and Li et al. (2020)’s study. First, we distinguished different extents of value change and creation but Li et al. (2020)’s study did not consider them. Second, Li et al. (2020) only selected woods as the raw material but we chose three materials in each experiment. Third, and most importantly, Li et al. (2020) have proposed the reason why the laborer modified the woods into a furniture (i.e., for marriage), but we did not mention this lest introducing confusing factors. All these differences might lead subjects’ responses more skewed toward the modifiers in Li et al. (2020)’s study, which need to be explored in future studies.

### 5.1. Limitations and implications

While our study addressed the role of transfer method in people’s ownership decisions between laborers and original owners, some limitations should be noted. First, this study takes ordinary adults as a sample to investigate the role of value change, creation and transfer types in labor-relevant ownership judgments. We did not determine whether young children and legal professionals would present similar reasoning on ownership issues. Due to their limited legal experience, children’s answers may better reveal the naive weighing of three elements during ownership resolution. Previous studies have found that young children place more emphasis on creative labor in ownership reasoning than adults (Kanngiesser et al., 2010, 2014; Faigenbaum et al., 2018), and they may display different response patterns in such research. The inclusion of professionals can help us directly compare the preferred solutions between lay people and legal scholars and find the root of possible inconsistency. Second, this study suggests that the intention of the original owner and the laborer might play an important role in third parties’ ownership assignments. Subjects were more likely to support the original owners when the laborers intentionally modified others’ objects without consent (i.e., in the *steal* and *borrow* conditions) but less likely to support the original owners when the laborers modified others’ objects without knowing that they were originally owned (i.e., in the *find* condition). We did not directly examine the effect of intent in the study. Future research may clarify this effect with a direct experimental design. Third, although we did not mention the relationship between the laborers and the original owners to avoid introducing confounding variables, different transfer methods may lead to different perceptions of the relationship between the laborers and the original owners (e.g., friends are more likely to take and borrow each other’s things without consent), which might introduce some unexpected effects to the results. Such scenarios should be strictly controlled in future studies. Finally, this study recruited Chinese adults as subjects. While the Chinese population is often regarded as a non-WEIRD (i.e., Western, Educated, Industrialized, Rich, and Democratic; Henrich et al., 2010) sample, the generalizability of the results needs to be tested with more cross-cultural research.

The results of this research will provide insights for real-life mediation of ownership conflicts. Because many complicated ownership cases involve transfer scenarios, this research indicates that people have different opinions in different transfer situations and these opinions may be contrary to the law. Although the law indicates that ownership of stolen/borrowed/found property cannot be transferred (Ma, 2003; Simeone, 2009), it did not consider the occasions when the property is modified which makes it appreciate greatly. At least in this study, we found that people did not absolutely support the original owner for found property. When mediating conflicts of ownership in real life, we should not only respect the rights of the original owners but also consider the interests of the creative laborers, especially when the laborers processed the objects inadvertently and increased the value of the objects greatly.

## Data availability statement

The raw data supporting the conclusions of this article will be made available by the authors, without undue reservation.

## Ethics statement

The studies involving human participants were reviewed and approved by Scientific Research Ethics Committee of Xi’an Jiaotong University. The patients/participants provided their written informed consent to participate in this study.

## Author contributions

ZL designed the study, collected and analyzed the data, and drafted the manuscript. DD revised the manuscript critically. All authors contributed to the article and approved the submitted version.

## Funding

This work was supported by MOE (Ministry of Education in China) Project of Humanities and Social Sciences (19YJC190005) and Fundamental Research Funds for the Central Universities, XJTU (SK2022035) and Research Project on Major Theoretical and Practical Issues of Philosophy and Social Sciences in Shaanxi Province (2022HZ1051).

## Conflict of interest

The authors declare that the research was conducted in the absence of any commercial or financial relationships that could be construed as a potential conflict of interest.

## Publisher’s note

All claims expressed in this article are solely those of the authors and do not necessarily represent those of their affiliated organizations, or those of the publisher, the editors and the reviewers. Any product that may be evaluated in this article, or claim that may be made by its manufacturer, is not guaranteed or endorsed by the publisher.

## Footnotes

^1^¥10 can buy approximately two subway tickets in China.

^2^¥10,000 can buy approximately two Thinkpad computers in China.

^3^Because Chinese characters are pictographs, writing is sometimes seen as an art, which may bring great value.
